# A post-marketing surveillance study on the safety and effectiveness of recombinant antithrombin gamma in patients with congenital antithrombin deficiency in Japan

**DOI:** 10.1186/s12959-026-00844-x

**Published:** 2026-02-27

**Authors:** Hidesaku Asakura, Manabu Iwabuchi, Masaki Fujita, Satoru Ito, Yuka Kanda, Hiroshi Kuwazawa

**Affiliations:** 1 Center for Thrombosis, Hemostasis and Vascular Diseases, Keiju Kanazawa Hospital, 6-26 Shimoshinmachi, Kanazawa-shi, Ishikawa, 920-0910 Japan; 2 Pharmacovigilance Division, Kyowa Kirin Co. Ltd., 1-9-2 Otemachi, Chiyoda-ku, Tokyo, 100-0004 Japan

**Keywords:** Antithrombin activity, Congenital antithrombin deficiency, Post-marketing surveillance, Pregnancy, Recombinant antithrombin gamma, Venous thromboembolism

## Abstract

**Background:**

Congenital antithrombin deficiency (CAD) is a serious inherited thrombophilia, classified as a rare disease. According to a study measuring antithrombin (AT) activity in the general Japanese population, approximately one in 650 people (0.15%) have AT deficiency. Deep vein thrombosis of the lower limbs is the most common clinical symptom. Pregnancy further increases the risk of venous thromboembolism in thrombophilia carriers. Recombinant human AT gamma (rhAT-gamma) is marketed for the treatment of thrombophilia due to CAD; however, no clinical trials for Japanese CAD patients have been conducted.

**Methods:**

Post-marketing surveillance was conducted at 22 sites in Japan from April 2016 to March 2022 to evaluate the safety and effectiveness of rhAT-gamma under clinical use conditions in CAD patients. Patients who had received rhAT-gamma for thrombophilia due to CAD were enrolled. The observation period for each patient was 12 months after treatment initiation or 28 days after the end of treatment.

**Results:**

During the study period, 28 patients were recruited. Of these, 22 patients (1 man, 21 women) comprised the safety and effectiveness population. The reasons for rhAT-gamma use overlapped, being surgery in 13.6% (3/22) of the patients, pregnancy in 86.4% (19/22), and others in 22.7% (5/22). rhAT-gamma was administered at 20–94 IU/kg once daily. In the safety evaluation, adverse events (AEs) and adverse drug reactions were observed in 31.8% (7/22) and 18.2% (4/22) of the patients, respectively. Serious AEs occurred in 9.1% (2/22) of the patients; no AEs resulted in death. In the effectiveness evaluation, rhAT-gamma was judged effective in all patients. AT activity increased in most patients, contributing to safe delivery outcomes in pregnant women. Although the data were limited, D-dimer levels decreased after rhAT-gamma administration.

**Conclusions:**

rhAT-gamma was safe and tolerable in Japanese CAD patients, with an increase in AT activity confirmed in most patients. Among pregnant women with a higher risk of thrombosis, no thrombosis was observed before or after delivery, leading to safe childbirth.

**Trial registration:**

University Hospital Medical Information Network; study ID, UMIN000050276; date of registration, 8 February 2023 (retrospectively registered).

**Supplementary Information:**

The online version contains supplementary material available at 10.1186/s12959-026-00844-x.

## Background

Congenital antithrombin deficiency (CAD) is the most serious and longest-recognized type of inherited thrombophilia [1], classified as a designated intractable/rare disease. The congenital deficiency of antithrombin (AT) increases the tendency to form thrombi, and serious thrombosis is possible in patients ≤ 40 years of age. According to a study measuring AT activity in the general Japanese population, approximately 1 in 650 people (0.15%) has AT deficiency [2]. AT deficiency is classified into two types: a quantitative deficiency type (Type I) and qualitative deficiency type (Type II). Type II has three subtypes based on the location of the mutations within the heparin-binding domain of AT [3]. The most common clinical symptom of AT deficiency is deep vein thrombosis of the lower limbs. Other symptoms include pulmonary thromboembolism, cerebral venous sinus thrombosis, and thrombosis of the mesenteric veins and upper limb veins. Thrombosis sometimes occurs spontaneously, but triggers, such as trauma, surgical invasion, pregnancy/childbirth, prolonged immobility, infection, and dehydration, are present in approximately 40% of patients [3, 4]. Pregnancy further increases the risk of venous thromboembolism (VTE) in thrombophilia carriers, and family studies of AT deficiency have reported that the absolute risk of pregnancy-associated VTE in individuals with AT deficiency is 16.6% (95% confidence interval: 0.0%–45.1%) [prenatal, 7.3% (1.8%–15.6%); postnatal, 11.1% (3.7%–21.0%)]) [5].

AT is a key regulator of the coagulation pathway and one of its most abundant components [1]. Therefore, AT supplementation can effectively restore normal hemostasis and reduce inflammation in patients with AT deficiency, particularly during hypercoagulable conditions, such as surgery or pregnancy [1]. The active ingredient in the medication (Acoalan^®^; Kyowa Kirin Co., Ltd., Tokyo, Japan) used in this study is recombinant human AT gamma (rhAT-gamma). This medication has a low infection risk and was developed as an alternative to human plasma-derived AT (pAT) preparations. rhAT-gamma was approved in Japan in 2015 for the treatment of thrombophilia due to CAD and disseminated intravascular coagulation associated with decreased AT. However, the clinical data package provided at the time of application for manufacturing and marketing approval included only a phase 1 trial (single-dose study) in CAD patients in Germany, Sweden, and the UK, with a very small sample size of 16 patients. Furthermore, no clinical trials of rhAT-gamma in Japanese CAD patients have been conducted.

In the present study, we conducted post-marketing surveillance to evaluate the safety and effectiveness of rhAT-gamma under clinical use conditions in CAD patients in Japan. Safety and effectiveness were assessed by identifying previously unknown adverse drug reactions (ADRs), characterizing the occurrence of ADRs, and considering factors influencing safety and effectiveness.

## Methods

### Study design and target population

This prospective, observational, post-marketing, surveillance was conducted at 22 sites in Japan in accordance with Good Post-marketing Study Practice. Patients who had received rhAT-gamma for thrombophilia due to CAD were enrolled during the registration period (from 1 April 2016 to 31 March 2020). The observation period for each patient was 12 months after treatment initiation or 28 days after the end of treatment, whichever occurred first.

### Administration of rhAT-gamma

The dosage and administration of rhAT-gamma were adjusted by the physician based on the package insert, which recommends a slow intravenous injection or drip infusion of 24–72 IU/kg once daily.

### Outcomes

#### Safety

We investigated the type and incidence of adverse events (AEs) and ADRs. AEs and ADRs were recorded by system organ class and preferred term using the Medical Dictionary for Regulatory Activities/Japanese version (MedDRA/J) v25.1. AEs and ADRs were counted only once in the tabulation, even if multiple AEs or ADRs with the same preferred terms were observed in the same patient. If causality could not be ruled out, the event was recorded as an ADR. Shock, anaphylaxis, hemorrhage associated with concomitant use of rhAT-gamma and anticoagulants, and hemorrhage from all causes were defined as safety specifications.

#### Effectiveness

The effectiveness of rhAT-gamma was evaluated by the investigators and each evaluation was based on clinical judgment and a three-level overall assessment (effective, ineffective, or undetermined), while consideration was given to the tendency for thrombosis, the treatment, and prevention of thromboembolism during the administration period.

### Statistical analysis

The safety analysis population was defined as patients who completed the survey form and did not meet any of the following exclusion criteria: cases outside the contract or registration period, cases not treated with rhAT-gamma, duplicate cases within the 1-year observation period after treatment initiation, cases with a history of rhAT-gamma treatment, or cases for which safety could not be evaluated. The effectiveness analysis population comprised patients from the safety analysis population, excluding those with unevaluable effectiveness data or off-label use. Statistical hypothesis testing was not conducted, and no imputation was made for missing data. SAS version 9.4 (SAS Institute Inc., Cary, NC, USA) was used for the statistical analyses.

## Results

### Patients’ characteristics

**Fig. 1:**
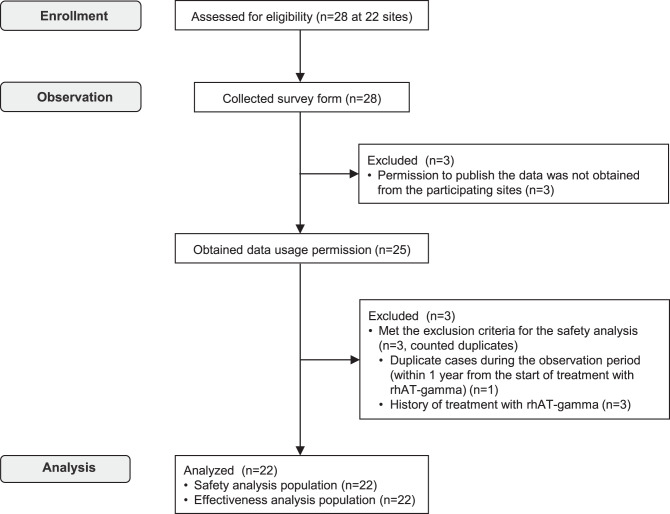
Patient disposition. Patients who received rhAT-gamma for thrombophilia due to CAD were enrolled. CAD: Congenital antithrombin deficiency; rhAT-gamma: Recombinant human antithrombin gamma

Between April 2016 and March 2020, 28 patients were recruited from the 22 participating study sites. Of these, three patients from three sites were excluded because these sites did not grant permission for the publication of their results in this study due to institutional policies, and three patients met the exclusion criteria for the safety analysis (one duplicate case during the observation period [within 1 year from the start of treatment with rhAT-gamma] and three with a history of treatment with rhAT-gamma). Some of these three cases were counted as duplicates; therefore, the safety analysis population comprised 22 patients. All 22 patients in the safety analysis population were included in the effectiveness analysis population (Fig. 1). Table 1 shows the patients’ characteristics at baseline. The study population comprised 4.6% (1/22) men and 95.5% (21/22) women. Of the women, 85.7% (18/21) were pregnant and 28.6% (6/21) were lactating. The median (minimum [min], maximum [max]) age was 31.5 (19, 57) years. The reasons for rhAT-gamma use overlapped, being surgery in 13.6% (3/22) of the patients (two patients with cesarean sections, one with missed abortion and evacuation of the uterus), pregnancy in 86.4% (19/22) of the patients, and others in 22.7% (5/22) of the patients (two patients with deep vein thrombosis, one with egg retrieval for in vitro fertilization in infertility treatment, one with pulmonary thromboembolism, and one with prepartum AT supplementation to prevent peripartum thrombosis). Prior medical therapy for thrombophilia or thromboembolism was administered in 68.2% (15/22) of the patients. The medications used for pretreatment were heparin sodium in 53.3% (8/15), warfarin potassium in 40.0% (6/15), heparin calcium in 40.0% (6/15), aspirin in 6.7% (1/15), and others in 20.0% (3/15). The median (min, max) AT activity was 42.0% (21.0%, 54.6%) at baseline.

**Table 1:** Patient characteristics

Item		Statistical parameter	
		n (%)	Median [min, max]
Total number of patients		22 (100.0)	
Sex			
Male		1 (4.5)	
Female		21 (95.5)	
Age (years)			31.5 [19, 57]
Body weight (kg)			55.7 [42.0, 88.0]
BMI (kg/m^2^)			22.3 [18.2, 33.1]
Reasons for rhAT-gamma use: (duplicates were counted)		-	
Surgery		3 (13.6)	
Pregnancy		19 (86.4)	
Others		5 (22.7)	
CAD category			
Type I		7 (31.8)	
Type II		3 (13.6)	
Subtype: Heparin-binding site		1 (33.3)	
Unknown		2 (66.7)	
Unknown		12 (54.6)	
Duration of disease (years)			
<1		8 (36.4)	
1 to <5		4 (18.2)	
5 to <10		4 (18.2)	
≥10		1 (4.5)	
Unknown		5 (22.7)	
History			
Allergy	Yes	6 (27.3)	
	No	16 (72.7)	
Thrombosis	Yes	8 (36.4)	
	No	14 (63.6)	
Other diseases	Yes	3 (13.6)	
	No	19 (86.4)	
Complication			
Thrombosis	Yes	3 (13.6)	
	No	19 (86.4)	
Hepatic dysfunction	Yes	1 (4.5)	
	No	21 (95.5)	
Others	Yes	8 (36.4)	
	No	14 (63.6)	
Pretreatment for thrombophilia or thromboembolism (pharmacotherapy)		15 (68.2)	
Heparin sodium		8 (53.3)	
Warfarin potassium		6 (40.0)	
Heparin calcium		6 (40.0)	
Aspirin		1 (6.7)	
Others		3 (20.0)	
Pretreatment for thrombophilia or thromboembolism (other therapies)		4 (18.2)	
Catheter-directed thrombolysis		1 (25.0)	
Venous stenting		2 (50.0)	
Physical therapy (exercise and compression)		1 (25.0)	
Others		2 (50.0)	
AT activity before administration (%)			42.0 [21.0, 54.6]
<50		18 (81.8)	
50 to <70		4 (18.2)	
Pregnant during the observation period		18 (85.7)	
Lactating during the observation period		6 (28.6)	

AT: Antithrombin; BMI: Body mass index; CAD: Congenital antithrombin deficiency; max: Maximum; min: Minimum; rhAT-gamma: Recombinant human antithrombin gamma

### Administration status of rhAT-gamma

Table 2 shows the administration status of rhAT-gamma. The median (min, max) initial daily dose and mean daily dose throughout the rhAT-gamma administration period were 36.0 (20.5, 72.0) and 40.5 (23.1, 93.3) IU/kg, respectively. The median (min, max) administration period and the number of days that rhAT-gamma was administered during that period were 31.5 (1, 220) and 8.0 (1, 211) days, respectively.

**Table 2:** Administration status of rhAT-gamma (safety analysis population)

Item	Statistical parameter	
	n (%)	Median [min, max]
Total number of patients	22 (100.0)	
Initial daily dose (IU/kg)		36.0 [20.5, 72.0]
<24	2 (9.1)	
24 to <36	7 (31.8)	
36 to <48	6 (27.3)	
48 to <60	3 (13.6)	
60 to ≤72	4 (18.2)	
Mean daily dose throughout the administration period (IU/kg)		40.5 [23.1, 93.3]
<24	1 (4.6)	
24 to <36	5 (22.7)	
36 to <48	7 (31.8)	
48 to <60	4 (18.2)	
60 to 72	4 (18.2)	
>72	1 (4.6)	
Administration period (days)		31.5 [1, 220]
<7	6 (27.3)	
7 to <14	3 (13.6)	
14 to <30	2 (9.1)	
30 to <90	2 (9.1)	
≥90	9 (40.9)	
Number of days rhAT-gamma was administered during the administration period		8.0 [1, 211]
<7	8 (36.4)	
7 to <14	6 (27.3)	
14 to <30	1 (4.6)	
30 to <90	6 (27.3)	
≥90	1 (4.6)	

max: Maximum; min: Minimum; rhAT-gamma: Recombinant human antithrombin gamma

### Safety

Table 3 shows a summary of the safety analysis results, indicating the AEs and ADRs. AEs and ADRs were observed in 31.8% (7/22) and 18.2% (4/22) of the patients, respectively. Although serious AEs and serious ADRs occurred in 9.1% (2/22) of the patients, neither AEs nor ADRs resulted in death.

**Table 3:** Safety summary of AEs and ADRs (safety analysis population)

Item	Number of patients n (%) [95% CI]	Number of events
Total number of patients	22 (100.0)	-
AEs	7 (31.8) [13.9–54.9]	8
Serious AEs	2 (9.1) [1.1–29.2]	2
AEs resulting in death	0 (0.0)[0.0–15.4]	0
ADRs	4 (18.2) [5.2–40.3]	4
Serious ADRs	2 (9.1) [1.1–29.2]	2
ADRs resulting in death	0 (0.0) [0.0–15.4]	0
Breakdown of ADRs	4 (18.2) [5.2–40.3]	4
System organ class		
Preferred terms		
Pregnancy, puerperium, and perinatal conditions	2 (9.1) [1.1–29.2]	-
Premature separation of placenta	1 (4.6) [0.1–22.8]	-
Intrapartum hemorrhage	1 (4.6) [0.1–22.8]	-
General disorders and administration site conditions	1 (4.6) [0.1–22.8]	-
Chest discomfort	1 (4.6) [0.1–22.8]	-
Investigations	1 (4.6) [0.1–22.8]	-
Platelet count decreased	1 (4.6) [0.1–22.8]	-
Breakdown of serious ADRs	2 (9.1) [1.1–29.2]	2
System organ class		
Preferred terms		
Pregnancy, puerperium, and perinatal conditions	2 (9.1) [1.1–29.2]	-
Premature separation of the placenta	1 (4.6) [0.1–22.8]	-
Intrapartum hemorrhage	1 (4.6) [0.1–22.8]	-

ADRs: Adverse drug reactions; AEs: Adverse events; CI: Confidence interval

The breakdown of ADRs was as follows: according to system organ class, “pregnancy, puerperium, and perinatal conditions” was reported in 9.1% (2/22) of the patients, while “general disorders and administration site conditions”, and “investigations” results accounted for 4.6% (1/22). Regarding the preferred terms, premature separation of the placenta, intrapartum hemorrhage, chest discomfort, and decreased platelet count were each reported in 4.6% (1/22) of the patients. Serious ADRs comprised premature separation of the placenta and intrapartum hemorrhage.

Table 4 shows the summary of the safety specifications. Shock, anaphylaxis, hemorrhage associated with concomitant use of rhAT-gamma with anticoagulants, and hemorrhage from all causes, were considered safety specifications. No AEs related to shock or anaphylaxis were reported during the study period, whereas AEs related to hemorrhage associated with concomitant use of rhAT-gamma with anticoagulants, and hemorrhage from all causes occurred in 9.1% (2/22) of the patients. The two patients who experienced hemorrhage from any cause were the same two patients who experienced hemorrhage associated with concomitant anticoagulant use. The observed AEs were premature separation of the placenta and intrapartum hemorrhage, both classified as serious ADRs.

**Table 4:** Summary of safety specifications

Item	Total AEs	Shock and anaphylaxis^a^	Hemorrhage associated with concomitant use of rhAT-gamma with anticoagulants^a^	Hemorrhage from all causes^a^
	n (%) [95% CI]	n (%) [95% CI]	n (%) [95% CI]	n (%) [95% CI]
Total number of patients in the safety analysis population	22 (100.0)	22 (100.0)	22 (100.0)	22 (100.0)
Number of patients with AEs^b^	7 (31.8) [13.9–54.9]	0 (0.0) [0.0–15.4]	2 (9.1) [1.1–29.2]	2 (9.1) [1.1–29.2]
Number of AEs	8	0	2	2
Seriousness				
Non-serious	6 (75.0) [34.9–96.8]	0	0 (0.0) [0.0–84.2]	0 (0.0) [0.0–84.2]
Serious	2 (25.0) [3.2–65.1]	0	2 (100.0) [15.8–100.0]	2 (100.0) [15.8–100.0]
Causal relationship with rhAT-gamma				
No	4 (50.0) [15.7–84.3]	0	0 (0.0) [0.0–84.2]	0 (0.0) [0.0–84.2]
Yes	4 (50.0) [15.7–84.3]	0	2 (100.0) [15.8–100.0]	2 (100.0) [15.8–100.0]
Factors other than rhAT-gamma				
No	2 (25.0) [3.2–65.1]	0	1 (50.0) [1.3–98.7]	1 (50.0) [1.3–98.7]
Yes	6 (75.0) [34.9–96.8]	0	1 (50.0) [1.3–98.7]	1 (50.0) [1.3–98.7]
Number of days from treatment initiation to AE onset (days)				
Median [min, max]	46.0 [1, 204]	-	136.0 [68, 204]	136.0 [68, 204]
Clinical outcome				
Recovered	5 (62.5) [24.5–91.5]	0	1 (50.0) [1.3–98.7]	1 (50.0) [1.3–98.7]
Improved	2 (25.0) [3.2–65.1]	0	1 (50.0) [1.3–98.7]	1 (50.0) [1.3–98.7]
Unknown	1 (12.5) [0.3–52.7]	0	0 (0.0) [0.0–84.2]	0 (0.0) [0.0–84.2]

^a^These AEs are all safety specifications. There were no important identified risks or missing information

^b^The denominator for calculating the percentage comprised the total number of patients included in the safety analysis population. The denominator for calculating the other percentages comprised the number of AEs in each category.

AEs: Adverse events; CI:Confidence interval; max: Maximum; min: Minimum; rhAT-gamma: Recombinant human antithrombin gamma

Of the ADRs reported in the present study, premature separation of the placenta and intrapartum hemorrhage were serious ADRs. These events were also ADRs related to the safety specifications of hemorrhage that is associated with concomitant use of rhAT-gamma with anticoagulants, and hemorrhage from all causes. The patient with premature separation of the placenta had a history of recurrent miscarriages. Factors other than rhAT-gamma such as infertility and the use of concomitant heparin were identified as causes of premature separation of the placenta. In the patient with intrapartum hemorrhage, heparin calcium had been used concomitantly with rhAT-gamma and prior to initiation of rhAT-gamma and was still being used when the AE occurred. rhAT-gamma was administered a total of 15 times in 74 days. The initial dose was 22.0 IU/kg, which was subsequently increased to a max of 70.0 IU/kg on the day of delivery. According to the investigator’s report, the AT activity may have increased during delivery because the dose was adjusted to target an AT activity of 75%–90%. Additionally, rhAT-gamma was administered twice on the day of delivery, and the patient went into labor after the second dose. Therefore, these factors may have caused heavy hemorrhage. Administration of rhAT-gamma was continued at a reduced dose, even after the AE occurred, and although the event was resolving 2 days after onset, a tendency for hemorrhage due to rhAT-gamma cannot be excluded.

### Effectiveness

Regarding effectiveness of rhAT-gamma, the investigators used the overall assessment to determine rhAT-gamma effectiveness in managing the tendency toward thrombosis and in treating and preventing thromboembolism during the administration period. rhAT-gamma was effective in 100.0% (22/22) of the patients at the time of the last assessment.

The time course of AT activity for each patient is shown in Supplement Fig. 1, divided by the reason for rhAT-gamma use. The changes in AT activity over time from the day of surgery or delivery are shown in Supplement Fig. 1A and B for patients who received rhAT-gamma for surgery and pregnancy, respectively, with the day of surgery or delivery as day 0. Supplement Fig.1C shows the time course of AT activity after treatment initiation (day 0) in patients with other reasons for rhAT-gamma use.

Of the three patients whose rhAT-gamma use was attributed to surgery, two underwent intrapartum cesarean sections, and the remaining patient underwent a surgery that did not involve delivery. Supplement Table 1 and Supplement Fig.1A show the AT activity changes for each of the three patients. In three patients for whom surgery was the reason for rhAT-gamma use, AT activity increased after initiation of rhAT-gamma administration. Of these patients, one patient (patient No. 3) showed a mild increase in AT activity. In this patient, rhAT-gamma was administered only on the day of surgery, and AT activity was 38.0% before administration and 42.0% 7 days after administration. For this patient, data were collected on the 7^th^ day after administration but not between the day of rhAT-gamma administration and this time point. The AT activity, which returned to near pre-administration levels by day 7 post-administration, is presumed to have increased in the interim.

The reason for rhAT-gamma use was pregnancy in 19 patients. Administration of rhAT-gamma was initiated from day −219 to day 0, and the median AT activity (min, max) before administration was 41.9% (21.0%, 54.6%). Supplement Table 2 and Supplement Fig. 1B show the AT activity changes for each of the 16 patients who were observed until the day of delivery (the remaining three patients were transferred to other hospitals, and observation was terminated before the day of delivery). The AT activity values immediately before delivery (AT activity after administration and on the day of or the day before delivery) were obtained in 12 of 16 patients, with AT activity in the 60% range, 70% range, and 80% range in three patients each, and 90% range, 120% range, and 130% range in one patient each. In 75% (9/12) of the patients, AT activity was sustained at or above 70%. In one patient (patient No. 6) for whom the reason for rhAT-gamma use was pregnancy, the AT activity before administration was 50.5%, and the max AT activity during the administration period was 45.6%. In this case, rhAT-gamma was initially administered at 30.0 IU/kg daily. The mean daily dose remained at 30.0 IU/kg throughout the 92-day administration period, with a total of 8 administrations and with no dose escalation. This suggests that insufficient administration of rhAT-gamma may have resulted in no increase in AT activity beyond baseline levels.

Other reasons for rhAT-gamma use were reported in five patients. The breakdown of the reasons is as follows: prevention of thrombogenesis in the peripartum period and AT supplementation before delivery, deep vein thrombosis with positive pregnancy reaction, egg retrieval for in vitro fertilization in fertility treatment, deep vein thrombosis, and pulmonary thromboembolism. Three of the five patients also received rhAT-gamma because of pregnancy. Supplement Table 3 and Supplement Fig. 1C show the AT activity changes for each of the five patients. In all five patients for whom the reason for rhAT-gamma use was reported as others, AT activity increased after rhAT-gamma administration.

**Fig. 2:**
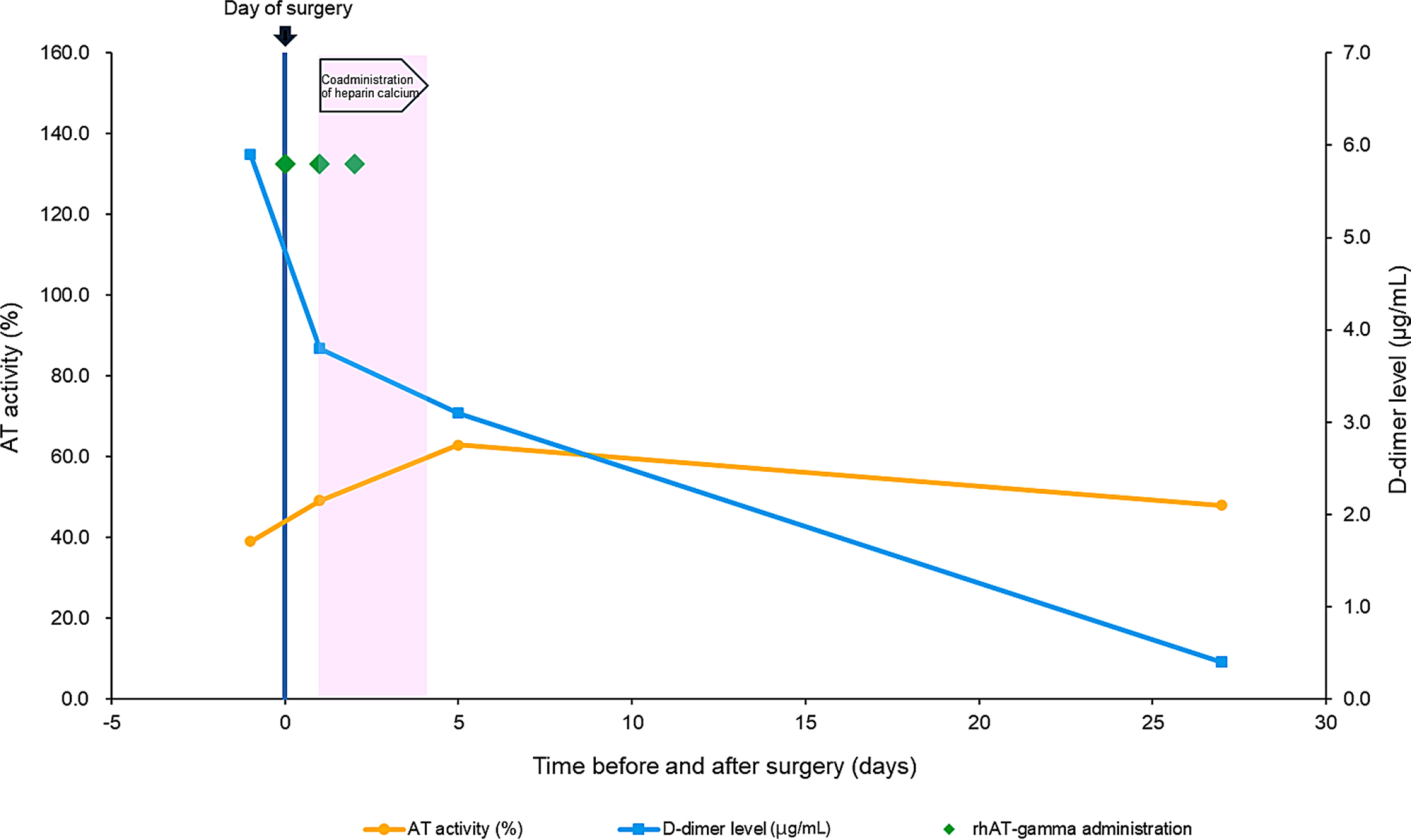
Time course of AT activity and D-dimer levels in patient No. 2. The patient received concomitant rhAT-gamma and anticoagulants, and the treatment effect of rhAT-gamma was confirmed on the basis of the D-dimer levels. The day of surgery was designated as day 0. AT: Antithrombin; rhAT-gamma: Recombinant human antithrombin gamma

**Fig. 3:**
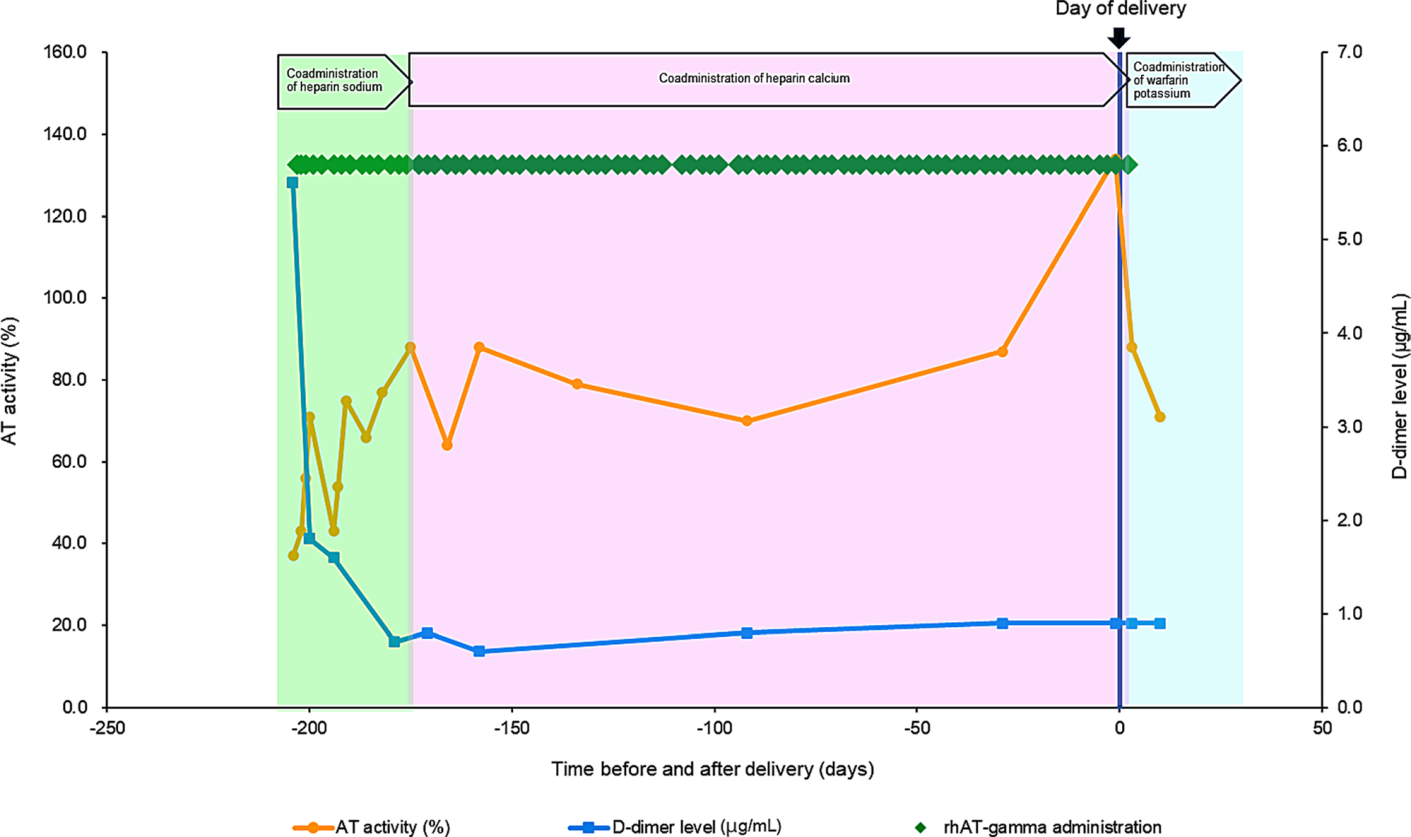
Time course of AT activity and D-dimer levels in patient No. 9. The patient received concomitant rhAT-gamma and anticoagulants, and the treatment effect of rhAT-gamma was confirmed on the basis of the D-dimer levels. The day of delivery was designated as day 0. AT: Antithrombin; rhAT-gamma: Recombinant human antithrombin gamma

Because insufficient data were available on the patients’ D-dimer levels, an overall assessment for this measurement could not be made in this study. For two patients in whom the treatment effect of rhAT-gamma was confirmed on the basis of D-dimer levels, the time course of AT activity and D-dimer level, and the administration status of rhAT-gamma and anticoagulants are shown in Figs. 2 and 3, respectively.

In patient No. 2, the reasons for rhAT-gamma use (CAD category: type II [heparin-binding site]) were surgery and pregnancy. The day of delivery (the day of surgery by cesarean section) was considered day 0, and rhAT-gamma was administered on days 0–2. The total daily dose of rhAT-gamma was 36.0 IU/kg. AT activity was 39.0% before administration, 49.0% on day 1, and 63.0% on day 5. The D-dimer level was 5.9 µg/mL before administration but decreased to 3.8 µg/mL the day after treatment initiation (day 1) and then decreased to 0.4 µg/mL 27 days after treatment initiation (day 27). Although heparin calcium was also administered, this therapy was initiated the day after rhAT-gamma was initiated. Therefore, the initial decrease in D-dimer level is likely attributable to the effect of rhAT-gamma. This suggests that treatment with rhAT-gamma improved the tendency toward thrombosis, enabling the patient to deliver safely by cesarean section.

In patient No. 9, the reason for rhAT-gamma use (CAD category: unknown) was pregnancy. rhAT-gamma was administered from day −203 to day 2 (number of days administered: 88 days), with the day of delivery as day 0. The initial daily dose and mean daily dose of rhAT-gamma were 28.6 and 62.0 IU/kg, respectively. AT activity was 37.0% before administration, whereas the median (min, max) during the administration period was 71.0% (43.0%, 134.0%). AT activity before the day of delivery was 134.0% of the max value. Although the D-dimer level was 5.6 µg/mL before administration (complicated with deep vein thrombosis at treatment initiation), the level decreased to 1.8 µg/mL 3 days after treatment initiation (day −200) and remained low thereafter. This patient had deep vein thrombosis at the time of treatment initiation. The D-dimer level was 5.6 µg/mL before administration and decreased rapidly to 1.8 µg/mL 3 days after treatment initiation, indicating an improvement in thrombosis tendency. rhAT-gamma administration continued during the subsequent pregnancy, and the median AT activity was maintained at the target level (70%). Additionally, AT activity just before the day of delivery was the highest value during the administration period, and no increase in D-dimer level was observed thereafter. This indicates that AT supplementation contributed to safe delivery, and that monitoring AT activity and D-dimer levels is crucial to prevent the onset and exacerbation of pregnancy-related VTE. Therefore, we recommend that both D-dimer levels and AT activity be monitored when administering AT preparations.

## Discussion

Acoalan^®^ is an rhAT-gamma preparation with a sugar chain structure similar to that of natural human AT preparations. rhAT-gamma was developed using Potelligent^®^ (Kyowa Kirin Co., Ltd.) technology [6], which relies on the Chinese hamster ovary host cell line to produce fucose-free recombinant proteins. Although native AT does not contain a fucose moiety, rhAT-gamma has structural similarity to that of native AT preparations and similar pharmacokinetic and biological activities to those of native AT. Currently, both freeze-dried concentrated human AT preparations and rhAT-gamma for intravenous infusion are covered by health insurance in Japan. To determine the optimal AT supplementation dosage, AT activity is evaluated before and after supplementation. Generally, concentrated formulations are typically administered at a standard single dose of 1000–1500 units, with the weekly administration frequency adjusted on the basis of the extent of the decrease in AT activity levels. rhAT-gamma can be produced stably without relying on human plasma donors [7], eliminating the risk of infection associated with pAT preparations. Furthermore, rhAT-gamma has shown similar safety and effectiveness to that at 1.2 times the dose of a human pAT preparation [8]. These features indicate that rhAT-gamma is a promising alternative to pAT products in terms of safety and effectiveness.

This study found that many of the patients enrolled in this study were prescribed rhAT-gamma for pregnancy-related thrombosis. However, even among pregnant women at high risk of developing thrombosis, no patients with thrombosis were noted before and after delivery, in this study, and all gave birth safely. In this study, rhAT-gamma was used not only as prophylactic therapy for thrombosis during pregnancy, but also for the treatment of a small number of patients with deep vein thrombosis and pulmonary thromboembolism. Notably, despite elevated D-dimer levels, further measurements were not conducted in some patients, leading to insufficient evaluation. Although limited, the available D-dimer data suggested a trend toward reduction following rhAT-gamma administration. Even when D-dimer data are insufficient, we believe that the AT activity results still provide a minimum level of confidence when interpreting effectiveness. However, further enlightening efforts are necessary to enable better early treatment with rhAT-gamma because D-dimer measurement remains limited in actual clinical practice.

This study showed that the incidence of ADRs was 18.2% (4/22), which might not be considered particularly low. However, 11 ADRs were observed in 31.3% (5/16) of the CAD patients in the Phase 1 trial, which did not involve Japan, and the major ADRs were pruritus and rash, which were reported in 2 patients (12.5%) each and not serious ADRs. As mentioned in the Results, serious ADRs related to hemorrhage were observed in two patients in this study, suggesting that appropriate AT activity monitoring is necessary, especially during delivery. In three patients for whom the reason for rhAT-gamma use was surgery, no abnormal hemorrhage was observed after surgery, and we confirmed that the surgery was completed safely.

In this study, the timing of AT activity measurement varied and was not specified in all patients, so no definitive comparisons could be made. However, an increase in AT activity was confirmed after rhAT-gamma administration. Overall, the investigators’ assessment judged rhAT-gamma effective in 100.0% (22/22) of the patients.

According to a survey of pregnant women between 2014 and 2018 in Japan, 27.5% of participants with AT deficiency had thrombosis [9]. Pabinger et al. showed that AT deficiency increased the tendency to develop thrombosis during pregnancy, and the incidence of thromboembolism was as high as 40% [10]. Additionally, a previous study demonstrated that women with thrombophilia had a higher risk of developing gestational VTE, regardless of whether there was a family history of VTE [11]. There are various reports regarding the incidence of VTE, but no definitive conclusions have been reached. The present study showed that the onset of VTE could be prevented by increasing AT activity through rhAT-gamma administration, suggesting that AT supplementation is a beneficial approach for reducing the risk of VTE. According to clinical guideline [12], supplementation with AT preparations is recommended during the perinatal period, depending on the individual VTE risk. Although there is no established consensus on AT supplementation, recent reports have recommended its use [13, 14]. Several studies have shown that AT activity should be maintained at least above 70%, and preferably above 80%, during delivery management [15]. Postpartum, in addition to basic heparin administration, supplementation therapy involves administering 1500–3000 units of pAT concentrate (or 1.2 times this dose for recombinant formulations) at delivery to achieve an AT activity of at least 70% and preferably above 80%. To postpartum day 5, maintaining AT activity above 70%–80% is the target, with the decision to administer based on AT activity and clinical symptoms [15, 16]. In this study, administration of rhAT-gamma was started from day −219 to day 0 (with the day of delivery as day 0), indicating that rhAT-gamma was administered to some patients in early pregnancy, while others received it shortly before delivery. Among 12 patients for whom predelivery AT activity (AT activity after the administration of rhAT-gamma and on the day of or the day before delivery) was measured, 75% (9/12) maintained AT activity above 70%, suggesting adherence to guideline-based delivery management.

This study has several limitations. First, the data were collected from a small population consisting solely of 22 patients in Japan. Second, the patients were skewed between men and women, with a ratio of 1:21, and 18 women were pregnant. One of the other three women began rhAT-gamma treatment on the day of delivery and was not classified as pregnant. This means that the reason for use was pregnancy in 19 patients. Therefore, most of the patients enrolled in this study were treated with rhAT-gamma for thrombophilia associated with pregnancy. However, we confirmed real-world use of rhAT-gamma not only for prophylactic administration against thrombus formation in conditions such as pregnancy but also as a treatment for established thromboembolic events, such as deep vein thrombosis and pulmonary thromboembolism. Although men were underrepresented in this study, the incidence rate of thrombosis does not differ significantly between men and women epidemiologically. Environmental factors for thrombosis risk include surgery, severe trauma, and pregnancy. However, many cases were triggered by pregnancy in this study. This likely resulted in the higher number of women participants. When used for surgery or severe trauma, rhAT-gamma is available regardless of gender. Finally, generalizability of the present findings beyond peripartum CAD patients is limited. Despite these limitations, we believe that the current results show that rhAT-gamma is the most appropriate alternative to pAT preparations in terms of safety and effectiveness.

## Conclusions

This study showed that rhAT-gamma was safe and tolerable in a real-world medical setting for patients with CAD in Japan. rhAT-gamma was effective, and increased AT activity was confirmed, regardless of the reason for rhAT-gamma use. Among pregnant women with a higher risk of thrombosis, no thrombosis was observed in early pregnancy or before and after delivery, leading to safe childbirth. Based on our analysis of real-world observational data, we conclude that administration of rhAT-gamma may decrease the risk of thrombosis in CAD patients.

## Electronic supplementary material

Below is the link to the electronic supplementary material.

Supplementary material 1

## Data Availability

Anonymized data obtained in this study and used for the analysis may be available upon reasonable request from a reader to the corresponding author.

## Abbreviations

ADRs: Adverse drug reactions
AEs: Adverse events
AT: Antithrombin
CAD: Congenital antithrombin deficiency
Max: Maximum
MedDRA/J: Medical Dictionary for Regulatory Activities/Japanese version
Min: Minimum
pAT: Plasma-derived antithrombin
rhAT-gamma: Recombinant human antithrombin gamma
VTE: Venous thromboembolism
