# Supplementary material for: A post-marketing surveillance study on the safety and effectiveness of recombinant antithrombin gamma in patients with congenital antithrombin deficiency in Japan
Source: Thromb J. 2026 Feb 27;24:32. doi: 10.1186/s12959-026-00844-x (PMC13003735; doi:10.1186/s12959-026-00844-x)
Supplement: Supplementary file 1 — Supplementary material 1 [file 12959_2026_844_MOESM1_ESM.pdf]

Supplement figure

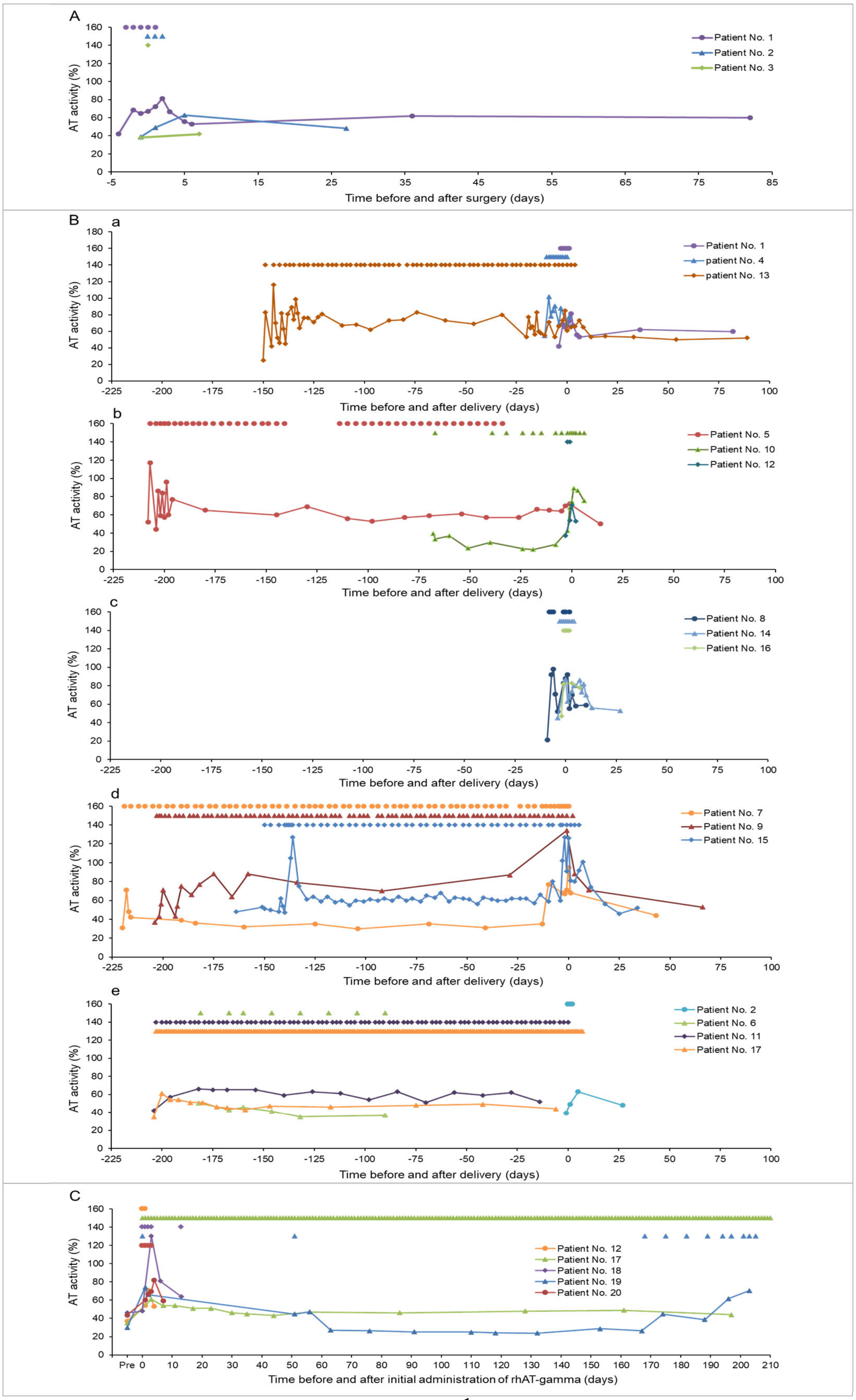

Supplement figure 1 Time course of AT activity in patients whose rhAT-gamma use was attributed to surgery (A), pregnancy (B), and other reasons (C).

The horizontal rows of dots above the line graphs indicate the timing of rhAT-gamma administration in each patient. The day of surgery was designated as day 0 on part A. Panels a-e of B show the results of patients with AT activity in the 60% range, 70% range, 80% range, ≥ 90% range, and no AT activity data immediately before delivery (after administration of rhAT-gamma and on the day of delivery or the day before), respectively. The day of delivery was designated as day 0 in B. The first day of administration was designated as day 0 in C. AT: Antithrombin; Pre: Pre-administration; rhAT-gamma: Recombinant human antithrombin gamma.

Supplement table 1 AT activity of patients whose rhAT-gamma use was attributed to surgery

Patient No.	Sex	Age (years)	Reason for use	Day of surgery (number of days from the start of administration, days)	Administration period (days)	Number of days rhAT-gamma was administered (days)	Initial daily dose (IU/kg)	Daily dose throughout the administration period (IU/kg)	AT activity (%)	
									Pre-administration	During the observation period<br><br>Median (min, max)
1	Female	20s	Cesarean section	4	5	5	36.0	26.4	41.9	65.8 (53.0, 81.2)
2	Female	30s	Cesarean section	1	3	3	36.0	36.0	39.0	49.0 (48.0, 63.0)
3	Female	30s	Missed abortion and evacuation of the uterus	1	1	1	38.0	38.0	38.0	42.0 (42.0, 42.0)

AT: Antithrombin; max: Maximum; min: Minimum; rhAT-gamma: Recombinant human antithrombin gamma.

Supplement table 2 AT activity of patients whose rhAT-gamma use was attributed to pregnancy

**Patient No.**	**Age, years**	**Day of surgery (number of days from start of administration, days)**	**Administration period (days)**	**Number of days rhAT-gamma was administered (days)**	**Initial daily dose (IU/kg)**	**Daily dose throughout the administration period (IU/kg)**	**AT activity (%)**		
							Pre-administration	After administration and on the day of delivery or the day before	During the observation period<br>Median (min, max)
1	20s	4	5	5	36.0	26.4	41.9	67.0	65.8 (53.0, 81.2)
2	30s	1	3	3	36.0	36.0	39.0	-	49.0 (48.0, 63.0)
4	30s	-	11	11	72.0	72.0	54.6	69.5	81.7 (68.3, 101.8)
5	20s	-	174	42	60.0	57.9	52.0	72.0	61.0 (44.0, 117.0)
6	20s	-	92	8	30.0	30.0	50.5	-	41.2 (35.0, 45.6)
7	30s	-	220	68	72.0	72.0	31.0	95.0	43.0 (30.0, 95.0)
8	30s	-	11	6	58.0	40.1	21.0	88.0	71.0 (52.0, 98.0)
9	20s	-	206	88	28.6	62.0	37.0	134.0	71.0 (43.0, 134.0)
10	20s	-	74	15	22.0	42.5	39.6	74.5	37.0 (21.9, 88.9)
11	20s	-	204	88	33.3	41.0	42.0	-	62.0 (51.0, 66.0)
12	30s	-	2	2	30.7	30.7	37.0	71.0	54.0 (53.0, 71.0)
13	30s	-	154	65	57.4	51.0	25.0	61.0	68.0 (42.0, 116.0)
14	30s	-	8	8	24.0	23.1	45.0	87.0	73.5 (53.0, 87.0)
15	40s	-	156	51	20.5	57.0	53.0	126.0	61.5 (46.0, 127.0)
16	30s	-	4	4	37.5	37.5	46.9	81.9	81.9 (77.8, 82.8)
17	30s	-	211	211	36.0	36.0	35.0	-	48.0 (43.0, 61.0)

AT: Antithrombin; max: Maximum; min: Minimum; rhAT-gamma: Recombinant human antithrombin gamma.

Supplement table 3 AT activity of patients whose rhAT-gamma use was attributed to other reasons

Patient No.	Sex	Age, years	Reason for use	Administration period (days)	Number of days rhAT-gamma was administered (days)	Initial daily dose (IU/kg)	Daily dose throughout the administration period (IU/kg)	AT activity (%)	
								Pre-administration	During the observation period Median (min, max)
12	Female	30s	Prevention of thrombogenesis in the peripartum period and AT supplementation before delivery	2	2	30.7	30.7	37.0	54.0 (53.0, 71.0)
17	Female	30s	Deep vein thrombosis with positive pregnancy reaction	211	211	36.0	36.0	35.0	48.0 (43.0, 61.0)
18	Female	10s	Deep vein thrombosis	14	5	30.0	30.0	46.0	72.5 (48.0, 130.0)
19	Female	30s	Egg retrieval for in vitro fertilization in fertility treatment	206	11	60.0	60.0	29.8	33.7 (23.9, 74.0)
20	Male	50s	Pulmonary thromboembolism	4	4	24.3	24.3	43.5	66.5 (59.0, 82.1)

AT: Antithrombin; max: Maximum; min: Minimum; rhAT-gamma: Recombinant human antithrombin gamma.
